# Two cases of type I sialidosis and a literature review

**DOI:** 10.1186/s13023-024-03431-3

**Published:** 2024-11-27

**Authors:** Yuan Ding, Ming Cheng, Chunxiu Gong

**Affiliations:** 1 Department of Endocrinology, Beijing Children’s Hospital, Capital Medical University, National Centre for Children’s Health, Genetics, Metabolism, Beijing, 100045 China; 2 MOE Key Laboratory of Major Diseases in Children, Beijing Children’s Hospital, Capital Medical University, National Center for Children’s Health, 56#Nan Lishi Rd, West District, Beijing, 100045 China

**Keywords:** Sialidosis, Alpha-N-acetyl neuraminidase, NEU1, Cherry-red spot, Muscle spasm

## Abstract

**Objective:**

This study aims to compare the clinical and electrophysiological characteristics of two cases of type I sialidosis in Chinese children with those reported in prior literature. The goal is to elucidate the clinical and genetic features of type I sialidosis.

**Methods:**

Clinical investigations and genetic analyses were conducted on an 11-year-old girl, primarily presenting with short stature, who was admitted in June 2020, and a 10-year-old boy, admitted in July 2023, exhibiting rapid weight gain and accompanying visual impairment as primary manifestations. A literature review was performed by summarizing data from 31 published articles encompassing 69 genetically confirmed cases of type I sialidosis up to 2023 for comparative analysis.

**Results:**

Patient 1 exhibited short stature, self-reported poor night vision, a history of occasional febrile seizures, mild scoliosis, bilateral cherry-red spots in the fundus, and prolonged P100 latency in both eyes as observed in visual evoked potentials (VEP). Genetic analysis revealed that she carried compound-heterozygous variants c.239 C > T (p.P80L) and c.880 C > T (p.R294C) in the NEU1 gene, inherited from her parents. Patient 2 presented with rapid weight gain and visual impairment, bilateral cherry-red spots in the fundus, abnormal neuroepithelial layer reflexes in both macular areas, approximately normal P100 latency but severely reduced amplitude in VEP after pupillary dilation, and severe bilateral optic nerve conduction block with relatively normal retinal cell function. Compound-heterozygous variants c.239 C > T (p.P80L) and c.803 A > G (p.T268C) were identified in the NEU1 gene of the Patient 2, inherited from his parents. By combining the cases reported in 31 literature articles with the 2 cases in our study, a total of 71 type I sialidosis patients were analyzed. The most common symptoms observed were muscle spasms (91.5%), followed by ataxia (75%) and seizures (63.6%). Intellectual impairment and abnormal electroencephalograms were more prevalent in Caucasian patients. Additionally, abnormal somatosensory evoked potentials, large cortical waves, and prolonged latency of VEP were more frequently observed in both Asian and Caucasian patients, serving as alternative indicators for early diagnosis.

**Conclusion:**

NEU1 gene analysis provides essential guidance for genetic counseling and prenatal diagnosis. The exon 2 variant c.239 C > T (p.P80L) in the NEU1 gene may represent a mutation hotspot among Chinese patients.

**Supplementary Information:**

The online version contains supplementary material available at 10.1186/s13023-024-03431-3.

## Introduction

Sialidosis is a hereditary metabolic disorder caused by mutations in the NEU1 gene. It is an autosomal recessive disease, classified into two types based on phenotype and age of onset. Sialidosis type I, also referred to as cherry-red spot myoclonus syndrome, is characterized by milder clinical symptoms, later onset, and manifestations such as ataxia, visual impairment, bilateral macular cherry-red spots, myoclonus, and seizures typically emerging in the second or third decade of life. In contrast, Type II, also known as the early-onset type (encompassing infantile and juvenile types), patients experience an earlier onset and a more severe clinical presentation. This includes facial dysmorphism, short stature, barrel-shaped chest, spinal deformities, skeletal dysplasia, intellectual disability, and hepatosplenomegaly. In the late stages of the disease, the characteristic cherry-red spots may disappear [1]. Additionally, some sialidosis type I patients may develop childhood cataracts. generally presents with a more severe phenotype, characterized by coarse facial features, hepatomegaly, multiple skeletal abnormalities, and delayed growth and development. Macular cherry-red spots are a typical ocular manifestation of this disease [2]. To date, only a few dozen cases of Type I sialidosis have been reported worldwide. This article presents the clinical diagnosis and treatment experience of two Chinese children with Type I sialidosis, supplemented by a literature review.

## Methods

### Subjects

We retrospectively collected clinical, laboratory and molecular data from two patients diagnosed with type I sialidosis. This study was approved by the Ethics Committee of Beijing Children’s Hospital. Parents of each patient were informed about the study procedure and provided informed consent prior to the collection of peripheral blood samples for whole-exome sequencing (WES). All detected variants were verified using Sanger DNA sequencing. The pathogenicity analysis of the identified variations was conducted in accordance with the guidelines of the American College of Medical Genetics and Genomics (ACMG) and cross-referenced with the 1000 Genomes Project database (http://www.1000genomes.org/).

## Literature review and statistical analysis

A comprehensive literature search was conducted using the keywords “type I sialidosis” and “NEU1” on PubMed, Wan Fang, and CNKI databases. Clinical phenotypes and NEU1 gene variations of patients with genetically confirmed type I sialidosis published from 1996 to 2023 were collated, with duplicate reports being excluded. The selected cases were summarized, and the clinical and genetic features among different ethnicities were compared.

Data were statistically analyzed using SPSS 22.0 software. Descriptive statistics were employed, with categorical data presented as counts and percentages, and continuous data expressed as means and ranges. Categorical data were represented as cases (%), and intergroup comparisons were conducted using the χ² test. A significance level of *P* < 0.05 was considered statistically significant.

## Results

### Case 1

#### Patient profile

A female patient, aged 11 years and 8 months, was admitted to our hospital in June 2020 with the primary complaint of short stature. The patient exhibited delayed physical development, with an average annual height increase of approximately 5 cm. There were no abnormalities in intelligence, dietary habits, or urinary and bowel functions. Over the past two years, the patient experienced easy fatigue, and her parents noted intermittent limb tremors during sleep. These episodes lasted approximately 1–2 min and resolved spontaneously, prompting no significant concern from the parents. Approximately two months prior to presentation, the patient experienced a seizure episode during a nap. This episode was characterized by limb shaking, limb stiffness, teeth clenching, bilateral eye deviation, frothing at the mouth, loud shouting, unresponsiveness, and lasted approximately 3–4 min. The local hospital considered the possibility of epilepsy and initiated treatment with neurotropic medication for one week prior to discharge. Subsequently, the patient experienced another seizure during trampoline activity, exhibiting the same symptoms as before. The patient was then prescribed oral levetiracetam and has remained seizure-free since initiation of this treatment. The parents reported that the patient experienced blurred vision at night, although daytime vision remained normal. Menarche occurred at the age of 11 with regular menstrual cycles and normal menstrual flow.

#### Birth and family history

The patient, born from a Gravida 2, Para 2 (G2P2) pregnancy, was delivered by a healthy mother who had regular prenatal check-ups. The birth occurred at full term without any history of perinatal asphyxia. Both parents are healthy, non-consanguineous, and have no family history of similar disorders. The patient’s 18-year-old brother is also healthy, with a height of 174 cm.

#### Physical examination

During the physical examination, the patient exhibited a height of 135 cm (less than the 3rd percentile) and a weight of 30 kg. Her body shape was symmetrical, and she displayed clear consciousness with appropriate responses. No skin abnormalities were observed. The assessment indicated Tanner stage III for bilateral breast development, steady breathing, and visible pectus carinatum. Additionally, the patient’s subcostal margins were slightly flared, although no bead-like changes in the ribs were detected. Respiratory sounds were clear, and the heart rate was measured at 88 beats per minute with a regular rhythm and no murmurs. There was no palpable liver or spleen. The examination also revealed mild scoliosis and X-shaped legs. Muscle strength was normal in all limbs, and bilateral Achilles tendon and knee reflexes were within normal limits. The Babinski sign was negative.

#### Auxiliary examinations

**Fig. 1:**
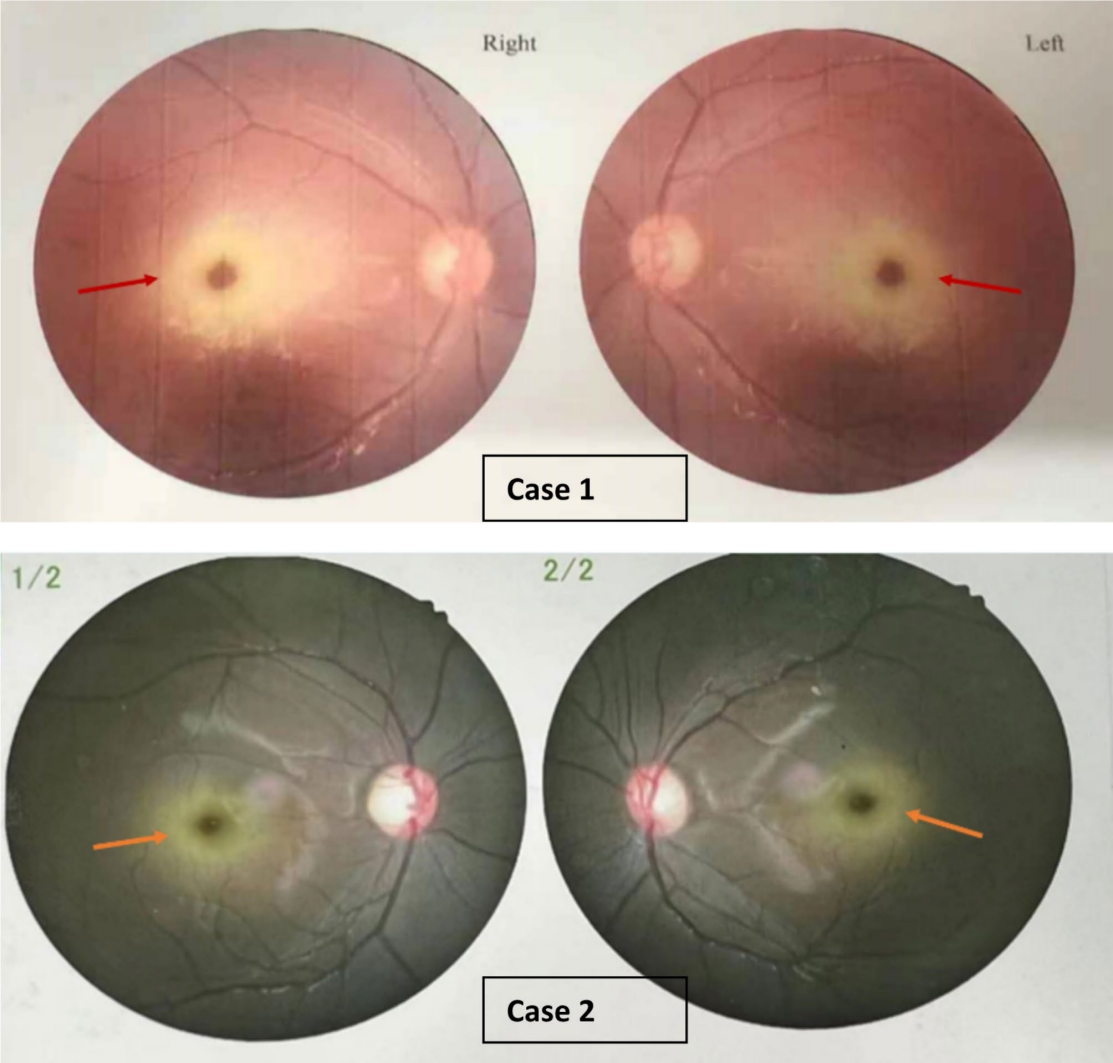
Cherry-red spots in the macular region of two patients with type I sialidosis

Routine blood, urine, and stool tests revealed no abnormalities. Serum electrolytes, as well as liver and kidney function tests, were within normal ranges. The amino acid and carnitine spectrum, along with urine organic acid analysis, showed no significant abnormalities. Imaging studies, including electrocardiogram, cardiac ultrasound, and head CT scan, indicated normal findings. Bone age assessment was consistent with approximately 13 years, although relatively short phalangeal segments were observed on the X-ray. he patient also exhibited spinal curvature, along with irregular vertebral body morphology in certain thoracic and lumbar vertebrae. A concave indentation in the central part of the vertebrae was observed in lateral views. Additionally, physiological curvature was noted. A 2-hour video electroencephalogram revealed no apparent abnormalities. The patient’s unaided vision was measured at 0.6 in the left eye and 0.5 in the right eye. Dilated correction visual acuity was recorded at 0.5 in the left eye and 0.3 in the right eye. Intraocular pressure was measured at 16 mmHg in the left eye and 17 mmHg in the right eye. Bilateral fundus examination revealed cherry-red spots (Fig. 1). The lens exhibited fine granular opacity. Visual evoked potential (VEP) examination demonstrated prolonged P100 latency in both eyes. Hearing assessments indicated normal hearing in both ears. Genetic analysis of the NEU1 gene identified compound heterozygous variations, c.239 C > T (p.P80L) [2] and c.880 C > T (p.R294C) [1], confirmed through WES (Supplementary Fig. 1). Both variations were inherited from the patient’s parents and have been previously reported as pathogenic. Consequently, the patient was diagnosed with type I sialidosis.

#### Treatment and follow-up

Given the absence of specific treatment options for Mucolipidosis (ML), symptomatic relief and nutritional support were implemented, with a focus on avoiding strenuous physical activity and protecting the joints. Regular ophthalmologic follow-up was also advised. At the current age of 14 years and 1 month, the patient continues to demonstrate normal intellectual and physical development, and is attending junior high school with excellent academic performance. However, intermittent seizure episodes persist.

### Case 2

#### Patient Profile

A 10-year and 7-month-old boy was admitted to our hospital in June 2023 with primary complaints of “rapid weight gain over the past 7 years and decreased vision for the past 2 years, which has worsened over the past half month.” The child has experienced accelerated weight gain since the age of 3, with a recent weight gain of 16.5 kg over the last year. The patient has a large appetite and prefers a diet rich in meat, fried foods, sweets, and beverages, while engaging in minimal physical activity. Vision decline began at the age of 8, without associated symptoms such as headaches, dizziness, or seizures. Despite wearing corrective glasses, the patient’s vision remained unclear as of mid-May 2023, with no other associated symptoms.

#### Birth and family history

The patient (G1P1), born to a healthy mother who underwent regular prenatal check-ups, was delivered at full term without complications, and there is no reported history of perinatal asphyxia. The patient’s younger sibling (G2P2), a 3-year and 5-month-old sister, is also in good health. His parents are non-consanguineous, and there is no family history of similar diseases.

#### Physical examination

The patient exhibited a weight of 69 kg, a height of 150 cm, and a BMI of 30.67 kg/m², surpassing the 95th percentile, which is indicative of nutritional excess and obesity without characteristic features of Cushing’s syndrome. The patient did not exhibit acne on the body but presented with acanthosis nigricans in the neck, axillary regions, and inner thighs. Additionally, striae, approximately 0.2 cm wide, were observed in the abdominal, buttock, and proximal thigh regions. Respiratory auscultation revealed clear lung sounds, and the cardiovascular examination indicated a heart rate of 92 beats per minute with a strong and regular rhythm, devoid of murmurs or additional heart sounds. No palpable liver or spleen was detected below the costal margin. The musculoskeletal examination demonstrated unrestricted joint extension, normal muscle strength and tone in all limbs, as well as normal bilateral Achilles tendon and knee reflexes, with a negative Babinski sign bilaterally.

#### Auxiliary examinations

Routine blood, urine, and stool analyses revealed no abnormalities. Serum electrolytes and renal function indicators were generally within normal ranges, with the exception of elevated alanine aminotransferase (ALT) at 128.6 U/L, aspartate aminotransferase (AST) at 70.1 U/L, and gamma-glutamyltransferase (GGT) at 37.7 U/L. The uric acid level was noted to be 420.9 µmol/L. A comprehensive glucose tolerance test conducted upon admission indicated a significant increase in insulin levels following glucose intake, suggestive of hyperinsulinemia. Analyses of blood amino acids, carnitine spectrum, and urine organic acids revealed no abnormalities. Normal findings were observed in electrocardiography, echocardiography, adrenal ultrasound, and cranial magnetic resonance imaging (MRI). Bone age was consistent with approximately 12 years. Sleep monitoring revealed the presence of obstructive sleep apnea and hypopnea syndrome. Pulmonary function tests indicated normal lung function. Pure tone audiometry and auditory brainstem response testing showed no abnormalities. Corrected visual acuity was measured at 0.6 + in the right eye and 0.7- in the left eye. Bilateral fundus examination revealed the presence of cherry-red spots (see Fig. 1). Abnormal reflexes were observed in the macular area. Visual evoked potential (VEP) testing reported a roughly normal P100 latency with severely reduced amplitude under low, medium, and high spatial frequency stimulation. Drug-induced pupil dilation visual evoked potential (VEP) testing revealed normal amplitude in both eyes under light and dark adaptation, indicating severe blockade of bilateral visual nerve conduction while suggesting overall normal retinal cell function. Genetic analysis identified compound heterozygous mutations in the NEU1 gene (c.239 C > T, p.P80L, and c.803 A > G, p.T268C), both inherited from the parents (Supplementary Fig. 1). These findings are consistent with previously reported pathogenic variants [1, 3]. Consequently, the patient was diagnosed with type I sialidosis.

#### Treatment and follow-up

The patient was advised to continue dietary management, adhering to a low-fat, calorie-restricted diet with a daily caloric intake limited to 1200 kcal. It is essential to intensify physical activity, engaging in moderate-intensity aerobic exercise for 40 min daily, aiming for active weight reduction at a rate of 2–2.5 kg per month. The patient was prescribed bicyclol and compound glycyrrhizin tablets for oral administration to support liver function and mitigate symptoms. Liver enzyme levels will be reevaluated at a scheduled follow-up, and medication adjustments will be made as necessary. Regular ophthalmological assessments were also recommended. Currently, the patient is in primary school, with average academic performance.

### Literature review

A comprehensive literature review identified a total of 1,342 articles. After excluding cases of type II sialidosis and those without confirmed genetic testing, we summarized 69 cases from 31 articles (detailed in Table 1). Among these cases, there were 32 male and 37 female patients. The average age of onset was 15.7 years (range: 5–33 years), and the average age at diagnosis was 24.1 years (range: 8–51 years). Including the two cases in this study, a total of 71 patients were analyzed. The most common initial symptoms were muscle spasm (91.5%), ataxia (75%), and epileptic seizures (63.6%), with many patients exhibiting more than one symptom. Among the 71 patients, 35 presented with cherry-red spots on the fundus. Intellectual impairment (22.9%, 11/48) and abnormal EEG findings (50.0%, 30/60) were more prevalent in Caucasian patients. Most patients experienced visual symptoms (66.2%). Brain MRI abnormalities were observed in 24 out of 58 cases (41.4%). Patient 1 in this study exhibited typical clinical features of type I sialidosis, including cherry-red spots on the fundus, muscle spasms, and epileptic seizures, but without ataxia. Cognitive function, EEG, and brain MRI were normal. However, she reported only blurred vision at night while maintaining normal visual acuity during the day. Patient 2 primarily presented with progressive vision loss and cherry-red spots on the fundus, but currently lacks other typical clinical features associated with type I sialidosis.

**Table 1:** Comparison of the clinical and electrophysiological characteristics of genetically diagnosed type 1 sialidosis patients in Asia and Europe

References	Number	Ethnicity	NEU1 mutation	Onset age	Average age at diagnosis	Gender (M/F)	Myoclonus	Ataxia	Epilepsy	Cherry-Red Spot	Cognitive impairment	Visual impairment	Abnormal EEG	Abnormal EP	Abnormal MRI
Caucasian (29 cases totally)															
Bonten et al., 2000 [1]	6	2 African- Americans 1 Greek 2 German 1 Dutch	p.R294S/p.L231H p.R294S/p.G218Ap.G227R (Homo) p.V54M/p.G378*	12.3 (8–17)	N.A.	1/5	5/6	4/6	4/6	4/6	1/6	3/6	2/6	N.A	N.A
Palmeri et al., 2000 [4]	1	Italian	Homo p.G328S	17	21	0/1	1/1	1/1	1/1	1/1	1/1	1/1	1/1	1/1	1/1
Sobral et al., 2014 [5]	1	Portuguese	p.D234N/p.R341*	26	53	1/0	1/1	1/1	N.A.	1/1	N.A.	1/1	1/1	1/1	1/1
Canafoglia et al., 2014 [6]	6	Italian	p.S67I (Homo) p.G227R/p.R305C	25.3 (22–31)	34.3 (27–42)	2/4	6/6	2/6	1/6	0/6	N.A.	2/6	5/6	6/6	1/6
Schene et al., 2016 [7]	2	Dutch	p.H399_Y400dup/p.E227R	12.5 (12–13)	14.5 (14–15)	0/2	2/2	2/2	0/2	2/2	N.A.	2/2	N.A.	N.A.	0/2
Ebru et al., 2016 [8]	1	Turkish	p.R305H/p.208del	17	N.A.	0/1	1/1	1/1	1/1	0/1	1/1	1/1	1/1	1/1	1/1
Mutze et al., 2016 [3]	1	German	p.S233R/p.Y268C	6	8	1/0	1/1	1/1	0/1	1/1	0/1	1/1	1/1	N.A.	0/1
Aravindhan et al., 2018 [9]	1	Ecuadorian	p.P210L (Homo)	16	39	1/0	1/1	1/1	1/1	1/1	0/1	1/1	1/1	1/1	0/1
Gultekin et al., 2018 [10]	1	Turkish	p.E209Sfs*94/p.D310N	18	24	1/0	1/1	1/1	1/1	1/1	N.A.	N.A.	N.A.	N.A.	1/1
Alaa S et al., 2019 [11]	1	Spanish	p.R294C (Homo)	9	25	1/0	1/1	1/1	0/1	0/1	1/1	1/1	N.A.	N.A.	1/1
Rossi S et al., 2020 [12]	1	Italian	p.L91R/p.G328S	13	28	0/1	1/1	0/1	1/1	1/1	N.A.	1/1	N.A.	N.A.	N.A.
Caciotti A et al., 2020 [13]	5	N.A	p.R294C/p.P335Q p.R294C/p.P335Q p.D177V/p.H337R p.L91R/p.G328S p.G227R/p.R280Q	12.3 (8–15)	N.A	0/5	4/5	3/5	3/5	5/5	N.A.	2/5	3/5	3/5	3/5
Vial F et al.,. 2022 [14]	2	Dutch	N.A.	13.5 (11–16)	N.A.	N.A.	2/2	N.A.	N.A.	2/2	N.A.	1/2	2/2	N.A.	N.A.
**Average**				15.8	28.8	9/20	27/29 (93.1%)	18/27 (66.7%)	13/26 (50.0%)	19/29 (65.5%)	4/11 (36.4%)	17/28 (60.7%)	17/24 (70.8%)	13/15 (86.7%)	9/20 (45.0%)
**Asian (42 cases totally)**															
Naganawa et al., 2000 [15]	2	Japanese	p.V217M/p.G243R	24.5 (17–32)	33.5 (25–42)	1/1	1/2	1/2	1/2	2/2	N.A.	1/2	N.A.	N.A.	0/2
Itoh et al., 2002 [2]	1	Japanese	p.P316S (Homo)	14	18	1/0	1/1	0/1	1/1	0/1	0/1	1/1	1/1	1/1	0/1
Lai et al., 2009 [16]	17	Chinese (Taiwan, China)	p.S182G (Homo) p.S182G/p.A319V p.S182G/p.Q55*	19.1 (12–33)	38.1 (25–51)	12/5	17/17	16/17	13/17	3/17	2/17	14/17	3/17	17/17	7/17
Ranganath et al., 2012 [17]	1	Indian	p.R294C/p.N398Tfs*90	12	14	0/1	1/1	1/1	N.A.	1/1	N.A.	N.A.	N.A.	N.A.	N.A.
Sekijima et al., 2013 [18]	1	Japanese	p.P80L/p.D135N	14	17	1/0	1/1	1/1	N.A.	1/1	1/1	0/1	N.A	N.A.	1/1
Gowda et al., 2017 [19]	1	Indian	p.G248C (Homo)	9	9	1/0	1/1	1/1	1/1	1/1	0/1	N.A.	0/1	N.A.	0/1
Hu et al., 2018 [20]	1	Chinese (Taiwan, China)	p.G248C (Homo)	12	15	1/0	1/1	1/1	1/1	1/1	1/1	N.A.	1/1	1/1	1/1
Mohammad et al., 2018 [21]	1	East Asian	p.S182G/p.G227R	12	20	0/1	1/1	1/1	1/1	0/1	1/1	0/1	0/1	N.A.	0/1
Ahn et al., 2019 [22]	1	Korean	p.R6Qfs*21/p.D310N	12	36	0/1	1/1	1/1	1/1	1/1	0/1	0/1	1/1	1/1	1/1
Fan et al., 2020 [23]	1	Chinese (Taiwan, China)	p.S182G/p.A106-G118del	5	12	1/0	1/1	1/1	1/1	0/1	0/1	0/1	1/1	1/1	0/1
^#^Liu MM et al.,2019 [24]	1	Chinese (Mainland China)	p.P80L/p.S182G	10	12	0/1	1/1	1/1	1/1	1/1	1/1	1/1	1/1	1/1	1/1
^#^Li YC et al., 2021 [25]	1	Chinese (Mainland China)	p.S182G (Homo)	12	25	0/1	1/1	1/1	1/1	N.A.	N.A.	1/1	1/1	1/1	0/1
Han X et al., 2020 [26]	4	Chinese (China’s mainland)	p.P80L/p.S182G p.R341X/p.S182G p.P80L/p.S182G p.D135N/p.R280X	10 (8–12)	16.5 (10–20)	2/2	3/4	3/4	3/4	3/4	0/4	4/4	2/4	2/4	3/4
Wang F et al., 2022 [27]	2	Chinese (China’s mainland)	c.544 A > G	12.5 (12–13)	N.A.	0/2	2/2	0/2	2/2	0/2	0/2	2/2	2/2	2/2	N.A.
Cao LX et al., 2021 [28]	1	Chinese (China’s mainland)	p.P80L/p.S182G	15	22	1/0	1/1	1/1	0/1	0/1	0/1	0/1	0/1	1/1	1/1
Neeraja K et al., 2021 [29]	2	Indian	p.R294C (Homo) p.R305P (Homo)	14	23.5 (14–33)	2/0	2/2	2/2	0/2	0/2	1/2	0/2	0/2	2/2	0/2
Rossi S et al., 2020 [30]	1	Chinese (Hong Kong, China)	p.L91R/p.G328S	13	28	0/1	1/1	N.A.	1/1	1/1	N.A.	1/1	N.A.	N.A.	N.A.
Li X, et al. 2020 [31]	1	Chinese (China’s mainland)	p.S182G (Homo)	9	11	0/1	0/1	0/1	0/1	1/1	0/1	1/1	N.A.	N.A.	0/1
Our study	2	Chinese (China’s mainland)	p.P80L/ p.R294C p.P80L/ p.Y268C	9.5	9.5	1/1	1/2	1/2	1/2	2/2	0/2	2/2	0/2	2/2	0/2
**Average**				15.1	27.1	24/18	38/42 (90.5%)	33/41 (80.5%)	29/40 (72.5%)	18/41 (43.9%)	7/37 (18.9%)	28/39 (71.8%)	13/36 (36.1%)	32/34 (94.1%)	15/38 (39.5%)
**Total**	71			15.4	27.6	33/38	91.5%	75.0%	63.6%	52.9%	22.9%	67.2%	50.0%	91.8%	41.4%
***P*** **Value**		0.631	0.643	0.025	1.000	0.136	0.005	0.341	0.424	0.074	0.011	0.755	0.745		

N.A., not available; EEG, electroencephalogram; EP, evoked potential. ^*^ represents a frameshift mutation; ^#^ represents Chinese literature. Non-normally distributed data are expressed as *M* (*Q*_*1*_, *Q*_*3*_); Comparisons of counting variables were performed by the Mann–Whitney *U* test, and categorical variables were performed by the Pearson’s chi-squared test or Yates’s correction for continuity

## Discussion

Sialidosis, also known as neuraminidase deficiency (MIM 26550), is a rare lysosomal storage disorder caused by mutations in the NEU1 gene, which encodes neuraminidase 1. This mutation results in reduced activity of α-N-acetyl neuraminidase, leading to impaired removal of terminal sialic acid residues from oligosaccharides, glycoproteins, and glycolipids in the lysosomes. This disruption in sialic acid degradation contributes to the pathogenesis of sialidosis [32]. Beyond its lysosomal function, NEU1 is also implicated in various metabolic processes, including cell proliferation and differentiation, immune and inflammatory responses, and lysosomal extracellular secretion [33]. Epidemiological data on the prevalence of sialidosis are scarce both domestically and internationally.

The NEU1 gene, located on chromosome 6p21.3, is associated with different clinical phenotypes of varying severity, depending on the specific type of mutation [1]. To date, over 30 NEU1 mutations have been identified as causes of sialidosis type I, with missense mutations being the most prevalent, while exon duplications or small deletions are less common [22]. In our study, the two cases revealed three mutations in the NEU1 gene (c.239 C > T, c.880 C > T, and c.803 A > G), all of which have been reported as pathogenic variants [1–3]. According to literature, the c.544 A > G mutation is a common missense variant among Taiwanese patients, with 88.2% of sialidosis patients exhibiting homozygous mutations. This variant is rarely reported in Caucasian patients, suggesting potential ethnic differences in the genotype and phenotype of sialidosis type I [16]. Among 12 mainland Chinese patients, 50% exhibited the c.239 C > T missense mutation in exon 2 of the NEU1 gene, indicating that this mutation may be a prevalent variant among this population. Our analysis revealed that the most common symptoms are myoclonus (91.5%), ataxia (75%), and seizures (63.6%). The onset age for sialidosis type I is typically between 14 and 15 years, with no statistically significant difference observed between European and Asian populations. Both sialidosis types are characterized by progressively worsening multifocal myoclonus, which generally manifests in the second decade of life and exhibits varying correlations with seizures and ataxia. Regardless of whether the condition is sialidosis type I or II, most patients in the later stages require lifelong wheelchair use due to severe movement disorders, primarily attributed to intense myoclonus. In comparison with existing literature, the onset age of the two cases in this study is earlier, approximately 10 years old, and the symptoms are milder. Notably, patient 2 presents only with progressive visual impairment without evident neurological involvement. Patient 1 exhibits nighttime blurred vision and seizures. Both patients demonstrate visual symptoms and cherry-red spots but have not yet shown the typical manifestations of myoclonus and ataxia. Laboratory examinations indicate delayed peaks in visual evoked potentials (VEP). Intellectual and physical development remains normal in both patients, necessitating long-term follow-up to monitor the progression of the clinical course.

Neuraminidase 1 (NEU1) is a lysosomal sialidase that cleaves terminal α-linked sialic acid residues from sialylglycans. NEU1 is biosynthesized in the rough endoplasmic reticulum (RER) lumen as an N-glycosylated protein to associate with its protective protein/cathepsin A (CTSA) and then form a lysosomal multienzyme complex (LMC) also containing β-galactosidase 1 (GLB1). Unlike other mammalian sialidases, including NEU2 to NEU4, NEU1 transport to lysosomes requires association of NEU1 with CTSA, binding of the CTSA carrying terminal mannose 6-phosphate (M6P)-type N-glycan with M6P receptor (M6PR), and intralysosomal NEU1 activation at acidic pH. In contrast, overexpression of the single NEU1 gene in mammalian cells causes intracellular NEU1 protein crystallization in the RER due to self-aggregation when intracellular CTSA is reduced to a relatively low level [34]. Mutations in the NEU1 gene can directly impact the active site or central region of sialidase, resulting in folding defects and causing sialidase to accumulate in the endoplasmic reticulum/Golgi apparatus. Additionally, mutations may affect the surface region of sialidase responsible for binding to the lysosomal multienzyme complex. Sialidase plays a pivotal role in the removal of terminal sialic acid residues from oligosaccharides and glycoproteins. Consequently, a deficiency in sialidase results in the accumulation of large sialic acid-rich molecules and the excretion of sialic acid oligosaccharides in urine [35]. Light and electron microscopy reveal cytoplasmic vacuolization in various cell types, including neurons, perineuronal and interfascicular Schwann cells, endothelial cells, and epithelial cells. This vacuolization is associated with diffuse cytoplasmic deposition of a lipofuscin-like pigment in neurons, which is detectable in several neural regions, such as the cerebral cortex, basal ganglia, thalamus, brainstem, spinal cord, among others [36]. Type II sialidosis is characterized by markedly low sialidase activity, whereas patients with the milder Type I variant may retain some residual enzyme activity. Furthermore, patients presenting with cherry-red spots may exhibit residual neuraminidase activity [21].

The laboratory diagnostic methods for sialidosis generally involve the detection of increased excretion of sialic acid in urine and the absence of neuraminidase activity in fibroblasts. Genetic analysis serves as a critical component for definitive confirmation. In this study, the patient’s diagnosis was corroborated through the identification of previously reported pathogenic variations in the NEU1 gene, in conjunction with the clinical manifestations observed. Our laboratory is currently unable to perform tests for sialic acid excretion in urine and neuraminidase activity in fibroblasts.

In the early stages of sialidosis, brain MRI findings may be unremarkable; however, as the disease progresses, manifestations such as cerebellar, pontine, and cerebral atrophy become evident. Muscle spasms in patients with sialidosis are often subtle yet rhythmic. Electroencephalography (EEG) typically reveals rapid discharge activity, making EEG-electromyography (EEG-EMG) correlation analysis a more reliable diagnostic method. This analysis consistently demonstrates a relationship between EEG spikes and myoclonic spasms. Patients with Type I sialidosis usually exhibit nearly normal EEG backgrounds, whereas those with Type II frequently present with multiple spike waves. Additionally, some patients display heightened evoked potentials and an enhancement of long-loop reflexes (LR or C reflex), which confirms significant cortical hyperexcitability contributing to muscle spasms. This intense rhythmic seizure activity reflects the characteristic features of long-loop reflexes triggered by midline neural stimulation [37]. Nearly all reported cases in electrophysiological studies involving evoked potentials demonstrate prolonged visual evoked potential (VEP) peak latency and abnormalities in somatosensory evoked potentials (SSEP), indicating that VEP and SSEP may serve as sensitive neurophysiological indicators for diagnosing Type I sialidosis, even in patients without visual symptoms. In the current study, Patient 1’s cranial CT scan and EEG examination revealed no notable abnormalities; however, regular follow-ups and, if necessary, cranial MRI are recommended. Patient 2 currently exhibits no neurological abnormalities, and EEG and head MRI examinations have yet to be conducted. In Patient 1, the visual evoked potential (VEP) peak latency is prolonged, which aligns with findings reported in the literature. Conversely, in Patient 2, while the VEP peak latency is generally normal, the amplitude is significantly reduced, a phenomenon also supported by existing literature. Sialidosis Type II typically manifests in infancy or early childhood with distinct facial and skeletal abnormalities, necessitating differentiation from other lysosomal storage disorders with similar phenotypic features. In contrast, Type I, primarily characterized by cortical myoclonus, must be distinguished from other progressive myoclonic epilepsy syndromes.

Patients with sialidosis and concurrent epilepsy are managed using pharmacological treatments similar to those employed for other Progressive Myoclonic Epilepsies (PMEs). PMEs constitute a group of disorders marked by myoclonic seizures, tonic-clonic seizures, and progressive neurodegeneration, frequently accompanied by cerebellar signs and dementia. Accurate diagnosis is crucial as it assists patients and their families in comprehending and coming to terms with the disease, despite its incurable nature [38]. The management of myoclonic and epileptic seizures in PMEs presents significant challenges, as these seizures often exhibit refractoriness to standard antiepileptic drugs [39]. Sodium valproate is often considered a first-line pharmacological agent, with severe myoclonus frequently necessitating adjunctive medications such as benzodiazepines, levetiracetam, piracetam, zonisamide, or topiramate. However, these treatments do not consistently yield effective results. Recent literature indicates that perampanel, a selective α-amino-3-hydroxy-5-methyl-4-isoxazolepropionic acid (AMPA) receptor antagonist, shows efficacy in the treatment of sialidosis. The precise mechanism underlying its anti-myoclonic effects remains not fully elucidated. Perampanel functions as a non-competitive antagonist at the AMPA receptor, which is believed to have a significant role in the pathophysiology of epilepsy. These receptors are implicated not only in the initiation of seizures but may also contribute to the progression of epilepsy. Consequently, perampanel can be considered as an adjunctive therapy for partial-onset seizures in patients with epilepsy and myoclonus [20, 40].

## Conclusion

Sialidosis is a rare, progressive lysosomal storage disorder. This study reports on the clinical, genetic, and neurophysiological characteristics of two patients from mainland China with Type I sialidosis and compares the onset characteristics of genetically confirmed Type I sialidosis patients in Europe and Asia over the past 30 years. Our findings indicate that rare cherry-red spots and prevalent NEU1 mutations are observed in Type I sialidosis patients in Asia. Abnormal somatosensory evoked potentials (SSEPs) accompanied by giant cortical waves and prolonged visual evoked potential (VEP) latency may serve as alternative markers for the early diagnosis of this disease. The p.P80L mutation is a common missense mutation observed in mainland Chinese patients. Due to limitations in laboratory conditions, the current study has certain constraints. Given the rarity of this disorder and the limited number of reported cases, future research should involve comprehensive clinical, electrophysiological, and genetic analyses, along with long-term follow-up, to achieve a more profound understanding of this rare condition.

**Fig. 2:**
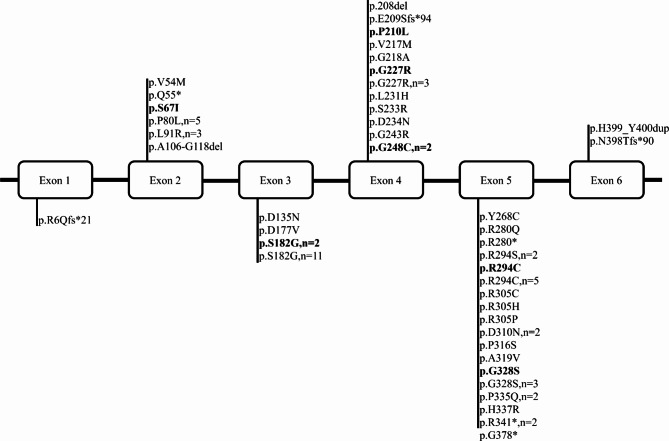
Genetic mutation spectrum of NEU1 gene in 71 patients with type I sialidosis (Bold font indicates homozygous mutations; “n” represents the number of cases.)

## Electronic supplementary material

Below is the link to the electronic supplementary material.

Supplementary Material 1: Partial genomic DNA sequences from the NRU1 genes of two patients and their parents

## Data Availability

To safeguard patient privacy, genetic sequencing data cannot be publicly disclosed. However, the information generated and analyzed in relation to the two cases discussed in this study is available from the corresponding author upon reasonable request.
