# Supplementary material for: Two cases of type I sialidosis and a literature review
Source: Orphanet J Rare Dis. 2024 Nov 27;19:440. doi: 10.1186/s13023-024-03431-3 (PMC11600752; doi:10.1186/s13023-024-03431-3)
Supplement: Supplementary file 1 — Supplementary Material 1: Partial genomic DNA sequences from the NRU1 genes of two patients and their parents [file 13023_2024_3431_MOESM1_ESM.pdf]

Supplementary figure 1. Partial genomic DNA sequences from the *NRU1* genes of two patients and their parents.

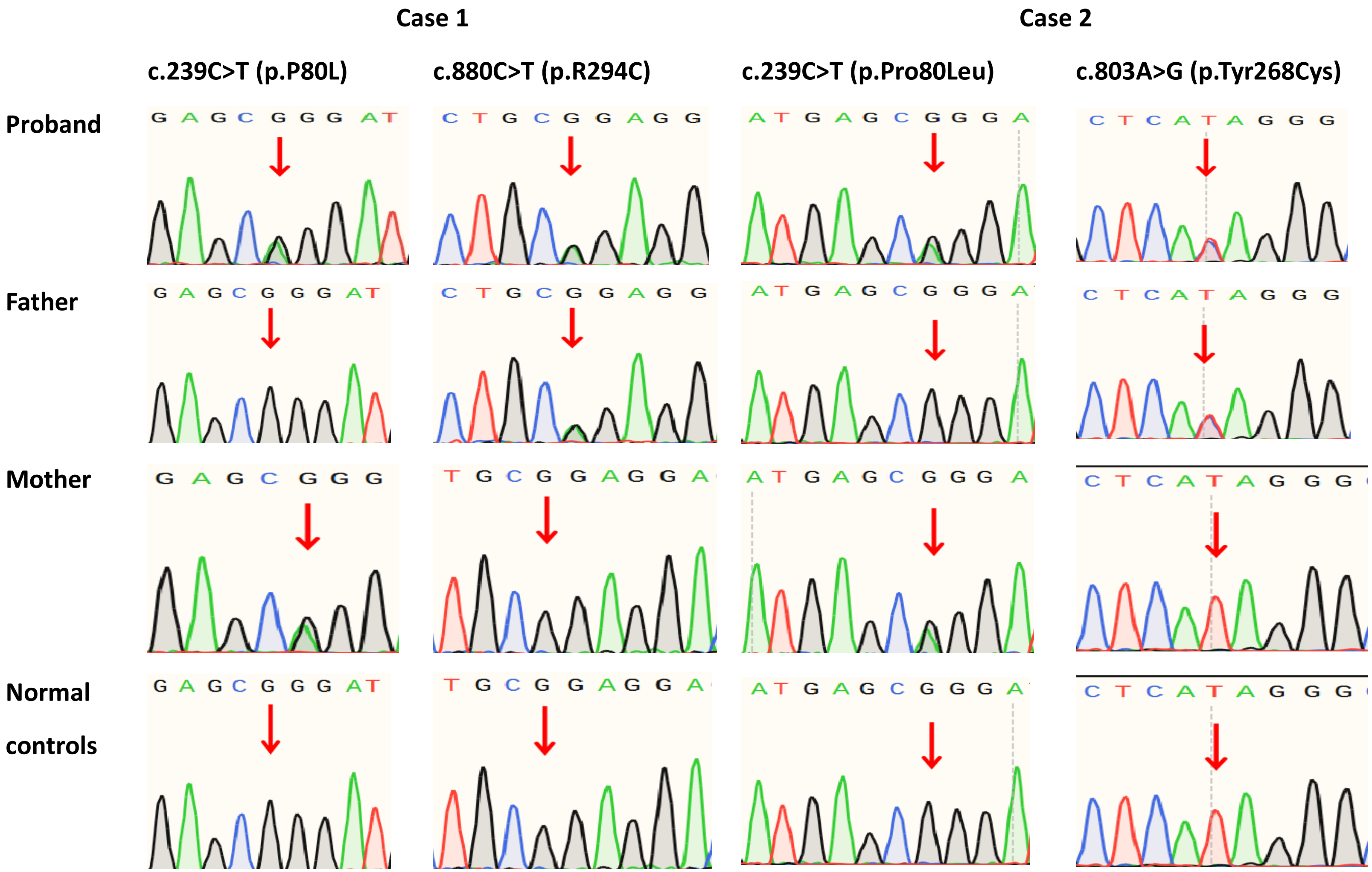

首都医科大学附属北京儿童医院医学伦理委员会

临床研究项目审查批件（IEC-C-006-A04-V.06）

伦理（科研）审编号：[2022]-E-062-Y

<table>
<tr><td>项目名称</td><td colspan="4">儿童肥胖脑功能研究</td></tr>
<tr><td>药物/器械/体外诊断试剂名称</td><td colspan="4">NA</td></tr>
<tr><td>临床试验分类</td><td colspan="4">药物适用： I期 □ II期 □ III期 □ IV期 □ 其他：科研
医疗器械 □ 体外诊断试剂 □</td></tr>
<tr><td>申办单位</td><td colspan="4">NA</td></tr>
<tr><td>CRO</td><td colspan="4">NA</td></tr>
<tr><td>牵头单位</td><td colspan="4">首都医科大学附属北京儿童医院</td></tr>
<tr><td>临床试验专业（科室）</td><td>内分泌遗传代谢科</td><td>主要研究者</td><td>巩纯秀</td><td>技术职称</td><td>主任医师</td></tr>
<tr><td>审查形式</td><td colspan="4">会议审查 ■ 快速审查 □</td></tr>
<tr><td>提交审查文件</td><td colspan="4">1. 临床研究项目伦理初审申请表
2. 研究方案（版本号：1.0，版本日期：20211214）
3. 知情同意书（版本号：1.0，版本日期：20211214）
4. 知情同意书（8岁以上儿童）（版本号：1.0，版本日期：20211214）
5. 参研人员资质证明</td></tr>
<tr><td>会议日期</td><td colspan="4">2022年2月22日</td></tr>
<tr><td>出席委员</td><td colspan="4">正式委员应到15人，实到8人；候补委员实到4人；回避0人，投票12人</td></tr>
<tr><td>投票结果</td><td colspan="4">同意10票， 必要的修改后同意（快速审查）2票，
必要的修改后同意（会议审查）0票， 不同意0票</td></tr>
</table>

审查意见

本伦理委员会的人员组成和工作程序符合ICH-GCP、中国GCP以及国家相关规定。

根据《赫尔辛基宣言》、《人体生物医学研究国际伦理指南》、《涉及人的生物医学研究伦理审查办法》、《药物临床试验质量管理规范》、《医疗器械临床试验质量管理规范》、《体外诊断试剂临床试验技术指导原则》、《儿科人群药代动力学研究技术指导原则》、《儿科人群药物临床试验技术指导原则》、《成人用药数据外推至儿科人群的技术指导原则》、《药物临床试验伦理审查工作指导原则》、《中医药临床研究伦理审查管理规范》和《中华人民共和国人类遗传资源管理条例》等的伦理原则，经本伦理委员会审查，同意该项目开展临床研究工作。

首都医科大学附属北京儿童医院医学伦理委员会
北京市西城区南礼士路56号
Tel: 01059616083; Fax: 01059616083; E-mail: bch_ec@163.com

CS 扫描全能王
3亿人都在用的扫描App

本批件将在各中心机构及其伦理委员会备案。

研究涉及采集、保藏、利用、对外提供我国人类遗传资源的情况，需向人类遗传资源管理办公室进行申报，获得批准后方可开展研究。

研究过程中若变更主要研究者，对临床研究方案、知情同意书、招募材料等的任何修改，请研究者提交修正案审查申请，获得批准后执行。

发生严重不良事件，请研究者及时提交严重不良事件报告。

请按照医学伦理委员会规定的年度/定期跟踪审查频率，研究者在截止日期前提交本中心研究进展报告。

研究纳入了不符合纳入标准或符合排除标准的受试者，符合中止试验规定而未让受试者退出研究，给予错误治疗或剂量，给予方案禁止的合并用药等没有遵从方案开展研究的情况；或可能对受试者的权益/健康以及研究的科学性造成不良影响等违背 GCP 原则的情况，请研究者提交违背方案报告。

研究暂停/提前终止，请研究者及时提交暂停/终止研究报告。

完成临床研究，请研究者提交结题报告。

年度/定期跟踪审查频率	12 个月	截止日期	2023年2月22日
批件有效期	1 年	截止日期	2023年2月22日

主任委员/副主任委员（签名）：[illegible]

2022 年 2 月 22 日

首都医科大学附属北京儿童医院

医学伦理委员会（盖章）

首都医科大学附属北京儿童医院医学伦理委员会

原件交由药物临床试验机构备案，复印件 3 份加盖公章：1、医学伦理委员会 2、申办方 3、研究者

CS 扫描全能王
3亿人都在用的扫描App
